# Natural history of Becker muscular dystrophy: a multicenter study of 225 patients

**DOI:** 10.1002/acn3.51925

**Published:** 2023-10-26

**Authors:** Akinori Nakamura, Tsuyoshi Matsumura, Katsuhisa Ogata, Madoka Mori‐Yoshimura, Eri Takeshita, Koichi Kimura, Takahiro Kawashima, Yui Tomo, Hajime Arahata, Daigo Miyazaki, Yasuhiro Takeshima, Toshiaki Takahashi, Keiko Ishigaki, Satoshi Kuru, Akiko Wakisaka, Hiroyuki Awano, Michinori Funato, Tatsuharu Sato, Yoshiaki Saito, Hiroto Takada, Kazuma Sugie, Michio Kobayashi, Shiro Ozasa, Tatsuya Fujii, Yoshihiro Maegaki, Hideki Oi, Hisateru Tachimori, Hirofumi Komaki

**Affiliations:** ^1^ Department of Neurology NHO Matsumoto Medical Center Matsumoto Japan; ^2^ Department of Neurology NHO Osaka Toneyama Medical Center Toyonaka Japan; ^3^ Department of Neurology NHO Higashisaitama National Hospital Hasuda Japan; ^4^ Department of Neurology National Center Hospital, National Center of Neurology and Psychiatry Kodaira Japan; ^5^ Department of Child Neurology National Center Hospital, National Center of Neurology and Psychiatry Kodaira Japan; ^6^ Department of Laboratory Medicine/Cardiology The Institute of Medical Science, The University of Tokyo Minato‐ku Japan; ^7^ Department of Information Medicine, National Center of Neurology and Psychiatry National Institute of Neuroscience Kodaira Japan; ^8^ Department of Clinical Data Science, Clinical Research & Education Promotion Division, National Center of Neurology and Psychiatry Kodaira Japan; ^9^ Department of Neurology, Neuro‐Muscular Center NHO Omuta National Hospital Omuta Japan; ^10^ Department of Medicine (Neurology and Rheumatology) Shinshu University School of Medicine Matsumoto Japan; ^11^ Department of Pediatrics Hyogo Medical University School of Medicine Nishinomiya Japan; ^12^ Department of Neurology NHO Sendai‐Nishitaga Hospital Sendai Japan; ^13^ Department of Pediatrics Tokyo Women's Medical University School of Medicine Shinjuku‐ku Japan; ^14^ Department of Neurology NHO Suzuka National Hospital Suzuka Japan; ^15^ Department of Pediatrics NHO Iou National Hospital Kanazawa Japan; ^16^ Research Initiative Center, Organization for Research Initiative and Promotion Tottori University Yonago Japan; ^17^ Department of Pediatric Neurology NHO Nagara Medical Center Nagara Japan; ^18^ Department of Pediatrics Nagasaki University Hospital Nagasaki Japan; ^19^ Department of Pediatrics National Rehabilitation Center for Children with Disabilities Itabashi Japan; ^20^ Department of Neurology NHO Aomori National Hospital Aomori Japan; ^21^ Department of Neurology Nara Medical University School of Medicine Kashihara Japan; ^22^ Department of Neurology NHO Akita National Hospital Yurihonjo Japan; ^23^ Department of Pediatrics Kumamoto University Hospital Kumamoto Japan; ^24^ Department of Pediatrics Shiga Medical Center for Children Moriyama Japan; ^25^ Division of Child Neurology, Department of Brain and Neurosciences, Faculty of Medicine Tottori University Yonago Japan; ^26^ Endowed Course of Health System Innovation Keio University School of Medicine Tokyo Japan

## Abstract

**Objective:**

Becker muscular dystrophy (BMD) is a milder variant of Duchenne muscular dystrophy (DMD), a lethal X‐linked muscular disorder. Here, we aim to investigat the clinical involvement of skeletal, respiratory, cardiac, and central nervous systems in patients with BMD, as well as genotype–phenotype relationships.

**Methods:**

This nationwide cohort study investigated the clinical manifestations and genotype–phenotype relationships in 225 patients with BMD having in‐frame deletion from 22 medical centers. The primary outcome was to elucidate the association of genotype with skeletal muscle, respiratory, cardiac, and central nervous system disorders. Descriptive statistics were used to analyze the data.

**Results:**

The average age of the subjects was 31.5 (range, 1–81) years. Initial symptoms of BMD were muscular (60%), followed by asymptomatic hypercreatine kinasemia (32.4%) and central nervous system disorders (5.3%). Gait disturbance was observed in 53.8% of patients and the average age at wheelchair introduction was 36.5 years. The ventilator introduction rate was 6.7% at an average age of 36.6 years. More than 30% of patients had an abnormal electrocardiogram and approximately 15% had heart failure symptoms. Cardiac function on echocardiography varied significantly among the patients. The frequencies of seizures and intellectual/developmental disability were 8.0% and 16.9%, respectively. Exon 45–47deletion (del) was the most common (22.6%), followed by exon 45–48del (13.1%). Patients with exon 45–49del patients demonstrated severe skeletal muscle damage. Patients with exon 45–47del and exon 45–55del patients did not require ventilator use.

**Interpretation:**

The study provides important prognostic information for patients and clinicians to establish therapy plans and to implement preventative medicine.

## Introduction

Becker muscular dystrophy (BMD), like Duchenne muscular dystrophy (DMD), is a dystrophinopathy caused by sarcolemmal dystrophin deficiency due to *DMD* gene pathogenic variants.^1^ BMD is known to present clinically from childhood to adulthood, with a high variability in symptoms, signs, and rate of disease progression.^1^ Most *DMD* variants causing BMD are in‐frame deletions, which are predicted to result in an internally deleted dystrophin protein that retains the amino and carboxy ends.^2^ Patients with BMD having in‐frame deletions of *DMD* often show a slower rate of clinical decline than those with DMD.^3^ If untreated and poorly monitored, cardiomyopathy of the patients with BMD can progress to heart failure, resulting in the need of heart transplantation.^4^ Early cardiac treatment can delay or slow the progression of cardiomyopathy, especially in patients with BMD having extra‐skeletal muscle involvement, although there have been cases of rapid deterioration of cardiac function due to delayed diagnosis caused by lack of awareness of rare neuromuscular diseases.^5^ Furthermore, patients with BMD, as well as DMD can develop central nervous system (CNS) disorders.^6^, ^7^ The association between the terminal portion pathogenic variants in *DMD* and CNS lesions has been demonstrated in DMD and BMD.^8^, ^9^, ^10^

BMD can be suspected based on hyperCKemia in neonates,^11^ leading to an increased number of diagnosed patients. However, the phenotype and severity of BMD vary widely, and the genotype–phenotype relationships and effects of early diagnosis on the prognosis of BMD are unclear. On the other hand, mini‐^12^ and micro‐dystrophin^13^ constructs for gene therapy for DMD are designed based on BMD genotypes with mild symptoms. In addition, exon‐skipping therapy for DMD can restore the translational reading frame to generate functional dystrophin proteins, such as in BMD. Therefore, it is important to clarify the natural history and the genotype–phenotype relationships in BMD.^14^ In this study, we conducted a study of the natural history of BMD that was started in 2017 as a project of the Muscular Dystrophy Clinical Trial Network (MDCTN). We explored the clinical manifestations and genotype–phenotype relationships of BMD to clarify disease progression for genetic counseling and preventative medicine.

## Methods

### Standard protocol approvals, registrations, and patient consents

This study was approved by the Institutional Ethical Review Boards of the National Hospital Organization Matsumoto Medical Center (approval no. 19–39) and all other participating institutes. All patients provided written informed consent to participate in the study. Case registration was centralized by the Muscular Dystrophy Clinical Trial Network (MDCTN). This study includes 20 patients with BMD reported in previous publications.^15^, ^16^

### Selection and enrollment of the participants

Selection of subjects for this study was based on having an in‐frame deletion mutation that did not include the 5′ and 3′ ends of the *DMD* gene,^17^ and being able to walk after the age of 16.^18^ In addition, to determine the natural history of patients under 16 years of age, patients who were considered to have BMD based on information from affected family members or phenotypes of BMD with the same in‐frame deletion were also included. This group included patients who had been introduced to a wheelchair but had partial use of it and were able to walk independently. Patients with duplications or small mutations such as nonsense or splice site mutations were excluded from the study. Patients affiliated with the representative or 21 cooperating research institutions between 13 June 2017 and 31 March 2020 were registered for the study. All patient data were extracted from medical records. Enrolled patients met the following inclusion criteria: (1) able to visit the institutions regularly (hospitalization was acceptable); (2) available medical records; and (3) patients (aged ≥16 years) or, for patient was <16 years, patient surrogates whose consent to participate in this study was obtained. A total of 225 patients were consented and enrolled. The research objectives, use of patient information, and the name of the information manager were displayed continuously on the websites of the research institutions and MDCTN to allow patients to withdraw from the study, if needed. The follow‐up period (from the time of initial medical record survey to the time of final medical record survey) ranged from 0 to 45 years, with a mean of 10.0 ± 7.2 years and a median of 9.0 years. Patients without at least a single follow‐up visit after diagnosis were excluded.

### Survey items

*DMD* pathogenic variants (in‐frame deletions) and clinical information obtained at both the initial and final medical record surveys were collected from medical records by a muscular dystrophy specialist. The medical record survey items included age, height, weight, *DMD* pathogenic variants, cardiac and CNS complications, initial symptoms or findings, gait status, serum CK levels measured using a spectrum altimeter at each facility, plasma brain natriuretic peptide (BNP) levels measured using a chemiluminescent immunoassay, serum cardiac troponin T (cTnT) levels measured using an electrochemiluminescent immunoassay, percentage of forced vital capacity [%FVC; measured vital capacity/predicted forced vital capacity (reference value)] according to age and gender (≥80% is the standard value), ventilator use, corticosteroid use, heart failure symptoms, electrocardiogram (ECG) findings, left ventricular diastolic diameter at end‐diastole (LVDd), and left ventricular ejection fraction (LVEF) measured using the Teichhloz method on echocardiogram, treatment for cardiomyopathy, and presence of CNS disorders.

### Statistical analyses

Survey items were analyzed using descriptive statistical analyses. The clinical characteristics recorded at the initial and final medical record surveys were compared. Kaplan–Meier survival curves were constructed for the deletion groups, and log‐rank test was used to compare the groups. Laboratory parameters recorded before and after the medical interventions were compared. Simple regression analyses were used to determine the correlations of laboratory data with age and between measurements from two timepoints. Missing values were excluded. The variables were compared between the genotype groups using violin plots and *t*‐tests. Log‐transformation was applied for serum CK and plasma BNP levels because of their expected log‐normal distributions.^19^, ^20^ Due to the large exploratory nature of the intergroup comparisons, *t*‐tests were conducted without adjusting for multiple comparisons. The (log) BNP, LVDd, and LVEF values were categorized according to use of cardioprotective agents; changes were visualized using violin plots. Data are presented as mean ± standard deviation (SD) or median (minimum–maximum). Statistical significance was set at *p* < 0.05. All statistical analyses were performed using R® (v. 3.5 or later; R Foundation for Statistical Computing, Vienna, Austria) and SPSS® (v. 23 or later; IBM Corp., Armonk, NY, USA) software. The study followed the STROBE reporting guidelines for cohort studies.

## Results

### In‐frame deletions in *DMD* gene of patients with BMD

**Figure 1:**
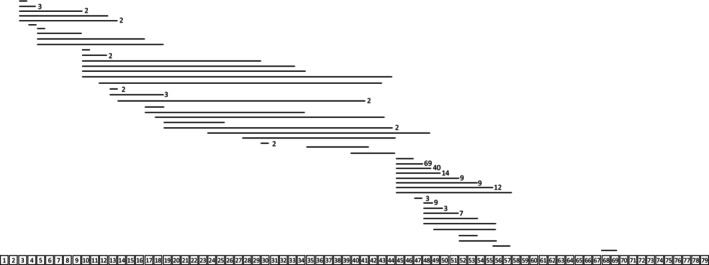
Types and distribution of the *DMD* gene in‐frame deletions in patients with BMD. The horizontal bars represent one or more exon deletions in the *DMD* coding region distributed over 79 exons. The numbers to the right of the bar represent the number of patients sharing deletions of the same exon. Bars with no number indicated have *n* = 1.

The patterns of in‐frame deletions of the 225 consented patients are indicated in Fig. 1. The first (exon 45–55 range) and second (exon 3–7 range) hot spots of *DMD* deletions accounted for 80.9% and 4.9% of all in‐frame deletions, respectively.

### Clinical characteristics of participants

Clinical characteristics of the 225 patients (age, 1–81 years; average age, 31.5 ± 17.9 years; mean age, 29 years) were shown in Table S1. Of the initial symptoms or findings that triggered patient diagnoses, skeletal muscle symptoms were the most common symptoms, followed by asymptomatic hyperCKemia, CNS, and cardiac complications.

Table S2 shows that 51.6% of participants had family members affected by BMD (61.2%) or an unknown diagnosis. While motor impairments were reported as initial symptoms in 30.2% of family members, other initial symptoms were similar to those of our participants (Table S2).

### Skeletal and respiratory muscle involvement and corticosteroid therapy

Gait disturbance was present in 53.8% of patients, of whom, 27.1% used a wheelchair (Table 1). Serum CK levels varied (Table 1 and Fig. S1A), and were in the normal range (<287 IU/L) in 3.6% (*n* = 8) (Fig. S1B) and 12.1% (*n* = 25) of the patients in the initial and final surveys, respectively. The genotype of the eight patients with normal CK levels at the initial survey was exon 3–4del, exon 10–34del, exon 17–34del, exon 45–46del, exon 45–48del, exon 45–55del, exon 49–55del, and exon 68–69del. In addition, the serum CK level at the initial survey was negatively correlated with age at wheelchair introduction (*r* = 0.729; *p* < 0.0001) (Fig. S1C).

**Table 1:** Skeletal and respiratory muscle involvement.

	*N* (%)	Mean ± SD (median, range)
All participants	225 (100)	
Gait disturbance at the time of survey	121 (53.8)	
Gait abnormality	47 (20.8)	
With device	13 (5.8)	
With wheelchair	61 (27.1)	
Age at introduction (years)		36.5 ± 15.8 (35, 9–72)
Part‐time use	15 (6.7)	
Full‐time use	36 (16.0)	

	*N*	
Serum CK		
At initial survey	212	
Age (years)		21.8 ± 16.9 (18, 0–73)
Value (IU/L)		3,582 ± 5,308 (1,480, 52–43,870)
At final survey	206	
Age (years)		30.7 ± 17.8*** (28, 0–81)
Value (IU/L)		1,783 ± 3,154*** (940, 22–30,850)
Mean value between two points	194	
Age (years)		26.4 ± 15.3 (25.5, 0–69)
Value (IU/L)		2,690 ± 3,445 (1,480, 118–30,529)
%FVC		
At initial survey	132	
Age (years)		28.7 ± 16.1 (28, 4–73)
Value (%)		91.5 ± 17.7 (91.5, 30.4–132.9)
At final survey	150	
Age (years)		34.8 ± 16.6** (34, 5–81)
Value (%)		90.6 ± 19.5* (91.2, 15.9–133.9)
Mean value between two points	124	
Age (years)		32.7 ± 16.3 (33, 4.5–77)
Value (IU/L)		91.6 ± 16.9 (92.3, 23.2–131.2)

	*N* (%)	
Use of artificial ventilator	15 (6.7)	
Night‐time only	7 (3.1)	
Intermittent (daytime)	1 (0.4)	
All day	6 (2.7)	
NPPV introduction	15 (6.7)	
Age at introduction		36.6 ± 14.2 (34, 18–61)
TPPV introduction	4 (1.8)	
Age at introduction		39.5 ± 13.7 (34.5, 30–59)

*p < 0.05; **p < 0.01; ***p < 0.001 versus mean value at initial survey; ns, not significant versus mean value at initial survey.

CK, creatine kinase; FVC, forced vital capacity; IU, international units; NPPV, non‐invasive positive pressure ventilation; SD, standard deviation; TPPV, tracheostomy positive pressure ventilation.

The %FVC values at the initial and final medical record surveys are presented in Table 1. These values did not correlate with age or age at wheelchair introduction (data not shown). Furthermore, no association was observed between serum CK level at the initial survey and age at ventilator introduction (data not shown). Ventilators were introduced in 6.7% of patients (Table 1).

There are no treatment criteria for BMD in Japan and the patients were administered corticosteroids at the discretion of the attending physician. Corticosteroids were administered to 10.7% of patients.

### Cardiac complications

The BNP and cTnT levels did not differ between surveys (Table 2) and did not correlate with age (data not shown). The BNP and cTnT values were higher than the upper limit of the normal range (18.4 pg/mL and 0.014 ng/mL, respectively) in 21.7% and 35.3% of patients, respectively, at the initial survey, and in 25.5% and 61.3% of patients, respectively, at the final survey.

**Table 2:** Cardiac complications.

	At initial survey	At final survey	Mean value between timepoints
Plasma cardiac biomarkers			
BNP (*N*)	160	184	151
Age [mean ± SD, (median, range)] (years)	26.2 ± 15.9 (24.5, 1–79)	32.6 ± 17.1*** (30, 0–81)	30.1 ± 16.3 (28, 4–80)
Level [(mean ± SD, (median, range)] (pg/mL)	47.0 ± 270.0 (7.1, 0.1–3283.0)	46.3 ± 208.6^ns^ (8.2, 1.2–2011.0)	51.5 ± 192.0 (10.2, 1.3–1697.5)
cTnT (*N*)	51	75	48
Age [mean ± SD, (median, range)] (years)	29.9 ± 17.6 (30, 1–79)	34.9 ± 16.3^ns^ (35, 7–81)	32.1 ± 17.3 (31.8, 4–80)
Level [mean ± SD, (median, range)] (ng/mL)	0.023 ± 0.0255 (0.018, 0.003–0.129)	0.022 ± 0.019^ns^ (0.017, 0.003–0.133)	0.023 ± 0.023 (0.019, 0.004–0.131)
Electrocardiography (*N*)	168	183	158
Age [mean ± SD, (median, range)] (years)	26.0 ± 16.9 (24, 0–73)	32.7 ± 17.1^ns^ (31, 4–81)	28.8 ± 17.0 (27, 3.5–77)
Heart rate [mean ± SD, (median, range)] (bpm)	79.3 ± 19.0 (45.5, 48–180)	73.5 ± 13.6** (73.5, 37–112)	77.0 ± 13.6 (74.3, 50.5–131.5)

Overall findings	*N* (%)	*N* (%)
Normal	61 (33.3)	61 (33.3)
Borderline	44 (26.2)	54 (29.5)
Abnormal	61 (33.3)	61 (37.2)
Basic rhythm		
Sinus rhythm	164 (97.6)	176 (96.2)
Sinus arrhythmia	4 (2.4)	7 (3.8)
Detailed findings		
Axis deviation	37 (22.0)	49 (26.8)
ST abnormality	46 (27.4)	38 (20.8)
T wave abnormality	27 (16.1)	37 (20.2)
Ventricular conduction abnormality	20 (11.9)	33 (18.0)
Suspected ischemic change	19 (11.3)	27 (14.8)
Left ventricular hypertrophy	20 (11.9)	16 (8.7)
Atrioventricular conduction abnormality	11 (6.5)	17 (9.3)
Abnormal *Q* wave	11 (6.5)	7 (3.8)
R/S > 1 in *V*_1_ lead	8 (4.8)	14 (7.7)
QT interval abnormality	8 (4.8)	6 (3.3)
Ventricular arrhythmia	7 (4.2)	5 (2.7)
Atrial load	4 (2.4)	5 (2.7)
Supraventricular arrhythmia	2 (1.2)	0 (0)

Echocardiography			
LVDd (*N*)	166	178	148
Age [mean ± SD, (median, range)] (years)	26.1 ± 16.7 (23.5, 0–73)	31.3 ± 16.7** (29, 4–77)	29.0 ± 16.2 (27, 3.5–73)
Value [mean ± SD, (median, range)] (mm)	45.8 ± 8.9 (45.0, 17.0–76.0)	47.3 ± 8.2^ns^ (46.2, 25.0–75.3)	46.7 ± 8.2 (45.6, 27.5–75.6)
Percentage of LVDd > 55 (mm)	12.6	14.1	12.2
LVEF (*N*)	178	174	153
Age [mean ± SD, (median, range)] (years)	26.0 ± 16.6 (23, 0–73)	31.6 ± 16.9** (29, 4–73)	29.3 ± 16.3 (27.3, 3.5–73)
Value [mean ± SD, (median, range)] (%)	56.9 ± 13.7 (60.4, 8.0–86.4)	55.3 ± 13.5^ns^ (59.1, 4.0–83.0)	55.9 ± 12.8 (59.0, 6.0–78.5)
Percentage of LVEF < 55 (%)	31.6	35.0	35.5

(During the entire survey period)	*N* (%)
Heart failure symptoms	34 (15.1)
Age [mean ± SD, (median, range)] (years)	34.9 ± 16.5 (36.5, 3–58)
Exertional dyspnea	12
Easy fatigability	6
Leg or generalized edema	4
Orthopnea	2
Others	11
Precursor of cold‐like symptoms	3
Use of cardiomyopathy drugs	101 (44.9)
Age at administration [mean ± SD, (median, range)] (years)	32.1 ± 15.0 (30, 3–73)
β–Blockers	62
Angiotensin converting enzyme inhibitors	58
Diuretics	14
Angiotensin II receptor blockers	8
Antiarrhythmic drugs	7
Cardiac stimulants	3
Antiplatelet drugs	2
Anticoagulants	2
Use of nondrug treatment	8 (3.6)
ICD	2
Cardiac pacemaker	1
CRT	1
Artificial heart device	1

**p < 0.01; ***p < 0.001 versus mean values at initial survey; ns, not significant versus mean value at initial survey.

bpm, beats per minutes; BNP, brain‐derived natriuretic peptide; CRT, cardiac resynchronization therapy; cTNT, cardiac troponin T; ICD, implantable cardioverter defibrillator; LVDd, left ventricular diastolic diameter; LVEF, left ventricular ejection fraction; *N*, number; SD, standard deviation.

The ECG findings are presented in Table 2. At both the initial and final survey, axial deviation, ST and T wave abnormalities, and ventricular conduction abnormalities were detected more frequently than abnormal Q wave and R/S > 1 in lead *V*_1_, which are considered specific for dystrophinopathies.

The LVDd at the initial survey prior to the administration of cardioprotective agents increased with age (*r* = 0.363; *p* < 0.0001), however, a total of 12.6% of patients had LVDd >55 mm (Table 2). The LVEF between the initial survey and the final survey was similar. The LVEF at the initial survey prior to the administration of cardioprotective agents correlated negatively with age (*r* = −0.383; *p* < 0.0001), but 31.6% of patients had LVEF < 55%. Among those patients, 40% were under 30 years old. Heart failure symptoms were present in 15.1% of patients, and 44.9% of patients received drugs and 3.6% received nondrug treatment for cardiomyopathy (Table 2).

With and without the use of cardioprotective drugs, no significant differences were observed between changes in plasma BNP, LVEF, and LVDd levels between the initial and final medical record survey (data not shown). Even when grouped by an LVEF cut‐off value of 55%, patients showed no significant difference in BNP levels at the initial and final surveys between groups. BNP values at both the initial and final surveys were not significantly different between with and without heart failure symptoms (data not shown). In‐frame deletions in patients with LVEF < 55% (*n* = 55) were distributed in the 5′ region and exons 45–55 of the *DMD* gene (Fig. S2A).

### CNS complications

The CNS complications observed are presented in Table 3. Patients with seizures (*n* = 19) and with intellectual/developmental disabilities (*n* = 39) commonly had in‐frame deletions in the middle or the second half (exon 45–55 range) of the coding region of the dystrophin rod domain (Fig. S2B,C, respectively).

**Table 3:** CNS complications.

	*N* (%)
Seizures	19 (8.4)
Febrile seizures	10 (4.4)
Epilepsy	10 (4.4)
Generalized seizures	2 (0.9)
Partial seizures	4 (1.8)
Other	1 (0.4)
Intellectual/developmental disorders	38 (16.9)
Intellectual disorder	16 (7.1)
Developmental disorder	12 (5.3)
ASD	7 (3.1)
ADHD	5 (2.2)
Psychiatric disorders	22 (9.8)
Adjustment/dissociative/panic disorders	9 (4.0)
Depression/bipolar disorder	9 (4.0)
Neurosis	7 (3.1)
Schizophrenia/delusional disorders	2 (0.9)

CNS complications were assessed by neuro‐pediatrics in children or by neurologist or psychiatry in adults.

ADHD, attention‐deficit/hyperactive disorder; ASD, autism spectrum disorder; CNS, central nervous system.

### Genotype–phenotype relationships in frequently occurring in‐frame deletions

**Figure 2:**
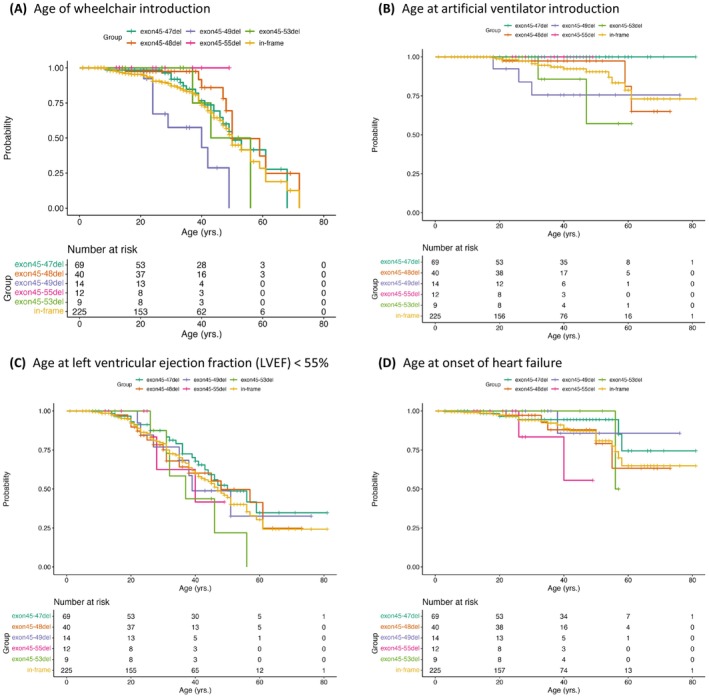
Kaplan–Meier analyses of all *DMD* in‐frame deletions and the five most frequent *DMD* in‐frame deletions. (A) Age at wheelchair introduction, (B) age at artificial ventilator introduction, (C) age at development of left ventricular ejection fraction (LVEF) < 55%, and (D) age at onset of heart failure. del, deletion; in‐frame, all in‐frame deletions. The curves show exon 45–47del (green), exon 45–49del (purple), exon 45–48del (orange), exon 45–55del (pink), exon 45–53del (yellow), and in‐frame (ochre). The bottom row of each graph shows the number of participants at risk in each group at the indicated intervals along the *x*‐axis.

We examined the genotype–phenotype relationships in five common in‐frame deletions that accounted for 64.0% of the deletions detected (Table 4). There was no significant difference between these five common deletions in terms of age and BMI. The serum CK level at the initial medical record survey was not significantly different between the deletion groups (Table 4, Fig. S3A). Serum CK levels did not change between the timepoints in any deletion group, regardless of corticosteroid use (Fig. S4A,B). Corticosteroid and wheelchair use were common in patients with exon 45–49del, but were absent in patients with exon 45–55del. Age at wheelchair introduction in patients with exon 45–49del was younger than those with exon 45–47del (*p* < 0.001), exon 45–48del (*p* < 0.001), and exon 45–55del (*p* < 0.01) (Fig. 2A).

**Table 4:** Phenotypes of five most frequent *DMD* in‐frame deletions (*n* ≥ 9).

	Exon 45–47del	Exon 45–48del	Exon 45–49del	Exon 45–55del	Exon 45–53del
Number of subjects (% in total)	69 (30.7)	40 (17.8)	14 (6.2)	12 (5.3)	9 (4.0)
Age at registration [mean, (95%CI)] (years) Range of age (years)	36.4 (32.0–40.8) 1–81	38.5 (33.5–43.5) 13–73	39.4 (31.5–47.4) 13–76	26.8 (18.9–35.3) 1–49	39.7 (29.8–49.5) 15–61
Body height at final survey [mean, (95%CI)] (cm)	165.7 (163.3–168.2)	165.7 (163.3–168.2)	161.4 (157.0–165.8)	156.3 (138.8–173.9)	166.5 (162.1–170.8)
Body weight at final survey [mean, (95%CI)] (kg)	54.1 (50.2–58.0)	59.1 (54.7–63.6)	54.0 (44.7–63.2)	53.8 (42.7–64.9)	61.8 (54.8–68.7)
BMI at final survey [mean, (95%CI)] (kg/m^2^)	20.6 (19.5–21.5)	21.4 (20.0–22.7)	20.5 (17.4–23.7)	20.9 (19.1–22.7)	22.9 (20.0–24.6)
Serum CK level at initial survey (mean, (95%CI)) (U/L) Age (years)	3983 (2672–5295) 24.9 ± 17.4	3194 (2124–4264) 29.1 ± 16.5	2514 (848–4182) 24.8 ± 16.4	4147 (1005–7290) 20.0 ± 16.2	3543 (1227–5859) 28.2 ± 15.2
Percentage of wheelchair usage [mean, (95%CI)] (%) Age at introduction (years)	31.9 (21.2–44.2) 40.7 ± 12.8	25.0 (12.7–41.2) 48.1 ± 14.5	57.1 (28.9–82.3) 31.6 ± 9.8	0 (0–0) NA	44.4 (13.7–78.8) 45.3 ± 7.9
%FVC					
At initial survey [mean, (95%CI)] Age (years)	98.1 (93.9–102.3) 31.9 ± 15.9	88.7 (82.4–95.0) 32.9 ± 16.1	92.2 (83.9–100.5) 31.0 ± 14.0	88.8 (83.5–94.1) 15.7 ± 7.3	91.4 (82.6–100.2) 31.3 ± 15.9
At final survey [mean, (95%CI)] Age (years)	95.3 (90.9–99.7) 40.0 ± 16.9	93.9 (88.7–99.1) 38.7 ± 15.3	90.5 (81.9–99.1) 40.5 ± 12.2	94.7 (86.6–102.9) 26.4 ± 8.9	95.0 (87.9–102.1) 40.1 ± 14.9
Percentage of ventilator use [mean, (95%CI)] (%) Age at introduction (years)	0 (0–0) NA	7.5 (1.6–20.4) 47.0 ± 13.0	21.4 (4.7–50.8) 24.7 ± 5.0	0 (0–0) (NA)	22.2 (2.8–60.0) 39.5
Percentage of corticosteroid use [mean, (95%CI)] (%) Age at administration (years)	5.8 (1.6–14.2) 31.7 ± 4.5	10.0 (2.8–23.7) 40.8 ± 11.1	14.3 (1.8–42.8) 4, 17	0 (0–0) NA	22.2 (2.8–60.0) 5, 42
Plasma BNP level (pg/mL)					
At initial survey [mean, (95%CI)] Age (years)	17.4 (2.0–32.9) 28.4 ± 17.1	52.3 (3.0–101.6) 29.8 ± 15.2	6.7 (4.2–9.2) 23.3 ± 11.6	8.1 (5.4–10.9) 25.8 ± 13.9	33.2 (−1.0–67.4) 33.2 ± 15.8
At final survey [mean, (95%CI)] Age (years)	26.2 (8.4–44.0) 38.2 ± 17.6	101.7 (−21.3–224.7) 36.1 ± 16.3	11.5 (5.2–17.8) 35.1 ± 12.2	9.0 (5.7–12.3) 24.3 ± 11.7	34.1 (−4.0–72.2) 39.6 ± 15.2
LVDd (mm)					
At initial survey [mean, (95%CI)] Age (years)	44.9 (42.8–46.9) 29.4 ± 16.6	49.2 (46.2–52.3) 32.1 ± 16.5	43.0 (37.8–48.1) 29.6 ± 15.7	52.7 (49.1–56.4) 29.3 ± 14.5	49.9 (44.5–55.3) 33.4 ± 14.0
At final survey [mean, (95%CI)] Age (years)	47.4 (45.3–49.6) 37.3 ± 16.7	50.6 (48.1–53.0) 34.4 ± 14.5	43.8 (40.1–47.5) 37.8 ± 13.5	49.9 (44.6–55.3) 27.6 ± 7.8	49.1 (42.9–55.4) 42.0 ± 14.7
LFEV (%)					
At initial survey [mean, (95%CI)] Age (years)	58.4 (55.1–61.6) 30.0 ± 16.4	52.2 (47.2–57.1) 31.7 ± 16.4	59.6 (54.5–64.7) 29.1 ± 15.5	54.3 (45.5–63.1) 29.3 ± 14.5	49.4 (33.8–65.0) 33.1 ± 13.9
At final survey [mean, (95%CI)] Age (years)	54.8 (50.8–58.8) 37.3 ± 16.7	52.7 (48.6–56.8) 35.6 ± 15.7	58.7 (54.3–63.2) 37.8 ± 13.5	59.4 (53.1–65.7) 25.6 ± 9.6	48.5 (35.6–61.4) 42.0 ± 14.7
Percentage of heart failure symptoms Age at initial survey (years)	8.7 35.0 ± 18.8	15.4 39.0 ± 12.0	14.3 38	16.7 26, 40	11.1 56
Percentage of cardioprotective drugs use [mean, (95%CI)] (%) Age at administration (years)	8.7 (3.3–18.0) 37.3 ± 16.2	15.0 (5.7–29.8) 29.6 ± 11.5	14.3 (1.8–42.8) 31.5 ± 13.6	16.7 (2.1–48.4) 28.3 ± 7.3	11.1 (0.3–48.2) 36.3 ± 13.3
Percentage of CNS complications [mean, (95%CI)] (%)	25.0 (16.2–36.5)	20.0 (10.2–35.0)	21.4 (6.8–48.3)	0 (0–0)	33.3 (11.7–64.9)

Ages shown in the lower row are presented as mean ± standard deviation.

BMI, body mass index; BNP, brain‐derived natriuretic peptide; CI, confidence interval; CK, creatine kinase; CNS, central nervous system; del, deletion; FVC, forced vital capacity; LVDd, left ventricular end‐diastolic diameter; LVEF, left ventricular ejection fraction; NA, not available.

The %FVC in patients with exon 45–47del was significantly higher than those with exon 45–48del and total in‐frame deletions (Table 4, Fig. S3B). Further, the %FVC in patients with exon 45–49del was significantly higher compared to those with total in‐frame deletions. Interestingly, no patient with exon 45–47del or exon 45–55del required ventilator support (Table 4, Fig. 2B). However, there was no significant difference in the change in %FVC values between the timepoints for any in‐frame deletion group, regardless of corticosteroid use (Fig. S4C,D).

Plasma BNP levels in patients with exon 45–47del at the initial medical record survey were significantly lower than those in patients with exon 45–48del and total in‐frame deletions (Table 4, Fig. S3C). The LVDd in patients with exon 45–48del was significantly larger than that with exon 45–47del and exon 45–49del, and the LVDd with exon 45–55del was larger than with exon 45–49del (Table 4, Fig. S3D). The LVEF was not different between the deletion groups (Table 4, Fig. S3E). Kaplan–Meier analysis showed no difference in age at the development of LVEF < 55% between the deletion groups (Fig. 2C), while the age of heart failure onset in exon 45–55del was younger than that in exon 45–47del (*p* < 0.05) (Fig. 2D). In exon 45–47del and exon 45–48del, BNP and LVDd were higher and LVEF was lower in the group treated with cardioprotective drugs than in the untreated group (Fig. S4E–J).

CNS complications were absent in our patients with exon 45–55del, whereas the incidence of CNS complications was >20% in patients with the other four in‐frame deletions involving exons 45–55 (Table 4).

## Discussion

This cohort study evaluated the natural history and major genotypes and phenotypes of 225 Japanese patients with BMD (age, 1–81 years). Genetic analyses of patients with BMD from six countries^15^, ^16^, ^21^, ^22^, ^23^, ^24^, ^25^ have revealed similar pathogenic variant types and frequencies. However, most analyses focused on patients with DMD. Only a single Japanese report included relatively simplified clinical data of patients with BMD (age, ≥17 years).^16^ The results of longitudinal medical, muscle strength, and timed function assessments in 83 patients with BMD having in‐frame deletions was reported recently as a prospective natural history study.^17^

The most common initial symptoms or findings that triggered a diagnosis of BMD were muscular, followed by hyperCKemia and CNS symptoms. Heart disease was the fourth most common initial presentation. Deletions in the first hotspot (i.e., exon 45–55 range) were reported previously in 63% of patients with DMD^26^ and in 80.9% of patients with BMD in our study, while deletions in the second hotspot (i.e., exon 3–7 range) were reported previously in 7% of patients with DMD^27^ and in 4.9% of patients with BMD in our study.

Although CK values were highly variable at both the initial and final medical record surveys, 3.6% of patients were in the normal range at the initial survey, suggesting that low or normal CK values may result in a missed diagnosis. However, there was a negative correlation between the CK levels at the initial survey and age at wheelchair introduction, suggesting that CK levels may be a prognostic factor in motor function in patients with BMD. Overall, age at wheelchair induction tended to be earliest in exon 45–49del and later in exon 45–55del and exon 45–48del. This difference may be due to differences in the three‐dimensional structure of the dystrophin protein.^28^

On the other hand, patients with exon45–47del maintained respiratory function, and none of those patients required ventilatory support. Only 5.8% of patients with exon45–47del received corticosteroids, and its effect was considered to be small. The dystrophin protein in exon45–47del has an abnormal fractional‐type three‐dimensional structure indicating a severe form,^28^ but this structural hypothesis may not be applicable for respiratory function.

Cardiac complication in BMD correlates with life expectancy and prognosis. In our data, echocardiography showed that 40% of patients with cardiac dysfunction were under 30 years old. Therefore, cardiac assessment should be conducted from an early age. Abnormal Q waves, R/S > 1 in *V*_1_ lead, and PR shortening on ECG are characteristic of dystrophinopathies,^29^ but <10% of our patients had these findings, whereas the frequencies of axial deviation, ST, T wave, and conduction abnormalities were high and increased with age. These ECG abnormalities may be a biomarker for the diagnosis of cardiomyopathy and may inform treatment.

BNP, LVDd, and LVEF values varied widely among patients with the same in‐frame deletion within exons 45–55, although there were differences in BNP and LVDd between some deletions, but not in LVEF. In patients treated with cardioprotective drugs, little change in BNP and LVEF levels between the two surveys was observed. This may reflect that patients treated with cardioprotective drugs were more cardiac dysfunctional in nature. The association between age and cardiac dysfunction on echocardiography was not significantly different between the in‐frame deletion groups, but was characterized by stronger myocardial impairment in exon 45–55del patients with mild skeletal and respiratory muscle impairment. However, patients in the exon 45–55del group were a decade younger than most other in‐frame deletions with few in number, which may be significant limiting factors when comparing this group to the others. The difference between organs is unlikely to be explained by the three‐dimensional structure of the dystrophin protein alone. For exon 45–55del, the relationship between its phenotype and potential modifiers has recently been examined and suggested that specific intronic breakpoints and LTBP4 haplotypes may be affected.^30^ Based on our results, phenotypic differences also exist within the same deletion such as exon 45–47del or exon 45–48del, and similar analyses will also be needed.

Patients with BMD and cardiac involvement carry frequent in‐frame deletions in the 5′ region and exons 45–55.^4^ In our study, most patients with LVEF < 55% had in‐frame deletions in the same regions. However, the possibility cannot be ruled out that the concentration of patients with deletions in this region may result in an apparently higher number of patients with cardiac complications. A previous study showed that patients with DMD having pathogenic variants containing the dystrophin isoform *Dp116* have less cardiac dysfunction.^31^ Despite differences between DMD and BMD, it was not possible to evaluate this hypothesis in BMD as there was only one patient with a mutation containing Dp116 (exon 68–69del).

It has been reported that the prevalence of epilepsy in Japan is 0.53% in children^32^ and 0.69% in adults.^33^ In comparison, the prevalence of BMD in Japan in our study was >6‐fold higher at 4.4%. Serum CK levels can be high immediately after an epileptic seizure, which may hinder a diagnosis of BMD. The prevalence of intellectual disability in Asia is estimated to be 0.06%–1.3%, similar to that in Western countries.^34^

Autism spectrum disorder (ASD)^35^ and attention‐deficit hyperactivity disorder (ADHD)^36^ are estimated to be prevalent 1.68% and 6.3% of the Japanese population, respectively. In our BMD patients, the prevalence of ASD (3.1%), but not ADHD (2.2%), was higher than that in the general population. No difference was observed in the prevalence of adjustment/dissociative/panic disorders or depression between the general population^37^ and our BMD patients. In our patients, CNS complications were predominantly found in in‐frame deletions in the region encoding the dystrophin rod domain. It has been reported that CNS involvement is associated with deficiencies in the dystrophin isoforms *Dp140* and/or *Dp71*.^38^, ^39^ As the promoter of *Dp140* and *Dp71* locate in intron 44 and 62, respectively, deletions after exon 45 may be associated with involvement of the isoforms, but other pathomechanisms may underlie in other deletions. It should be noted, however, that we have not ruled out the possibility that CNS disorder in our patients with BMD are caused by factors other than *DMD* pathogenic variants.

Exon‐skipping therapies have been developed for DMD, targeting exons 51,^40^ 53,^41^ and 45,^42^ and others. The number of patients with BMD having in‐frame deletions suitable for exon‐skipping therapy is presented in Table S3. Our data may also be useful for the practice and care of patients after exon‐skipping therapy.

## Limitations

The study included a wide range of patients, including young pre‐onset cases, and phenotypes may be influenced by a variety of factors, including exercise, corticosteroids, rehabilitation, obesity, and orthopedic disease. Further, the data collected from patients diagnosed long ago may not be comparable to those from recently diagnosed patients, and differences in follow‐up durations may also affect the results. Therefore, multivariate analysis using these data is difficult. Because patients were included from 22 institutes, heterogeneity among specialists and institutes, data entry errors, missing or incomplete information, confounding factors, and information bias may have affected the results. Additionally, data from multigroup trials may be biased and subject to false positives or false negatives due to study design, small sample size, and multiple comparisons. Therefore, we are conducting a prospective study to validate the findings of this study.

## Conclusions

Our findings showed that the degree of skeletal and respiratory muscle impairment varies greatly depending on the type of in‐frame deletion, particularly in the severe impairment in patients with exon 45–49del and no ventilator users in the exon 45–47del and exon 45–55del patients. On the other hand, myocardial impairment was not clearly associated with the degree of deletion‐based skeletal muscle impairment. The prevalence of CNS complications was significantly higher than the general prevalence, but did not differ among the major deletion mutations. The results of this study may provide a more comprehensive natural history of BMD. In addition, our data may stimulate new studies on the structure and function of dystrophin proteins, dystrophin isoforms, and other epigenetic factors involved in dystrophinopathies.

## Author Contributions

A.N. had full access to all the data in the study and was responsible for the integrity of the data and the accuracy of the data analysis; A.N., T.M., K.O., M. M‐Y., E.T., K.K., and H.K. contrbuted to conception of the study; A.N., H.O., and H.T. contributed to the statistical analysis plan; T.K., Y.T, and H.T. contributed to statistical analysis; all authors contributed to the acquisition, analysis, or interpretation of the data; A.N. and H.T. contributed to drafting the text of the manuscript; A.N., H.K., and T.M. obtained fundings; H.O. and H.K. contributed to administrative, technical, or material support.

## Conflict of Interest

The authors report no conflicts of interest regarding this study.

## Supporting information


**Figure S1.** Serum creatine kinase (CK) levels at the initial medical record survey. (A) Scatterplot showing the association between serum creatine kinase (CK) level and age at the initial survey in all participants (*n* = 212). (B) Scatterplot showing the association between serum CK level and age at the initial survey with cut of level of 1000 IU/L; dash line shows upper limit level of normal range (287 IU/L). (C) Scatterplot of the association between serum CK at the initial survey and age at wheelchair introduction (*n* = 54); dash line linear indicates the regression derived from data.

**Figure S2.** Distribution of the *DMD* gene mutations in each phenotype. Bars represent one or more exon deletions in the *DMD* coding region distributed over 79 exons. (A) Left ventricular ejection fraction (LVEF) < 55% (*n* = 55); (B) seizures (*n* = 19); and (C) intellectual/developmental disability (*n* = 39). The numbers to the right of the bar show the number of patients. DMD, Duchenne muscular dystrophy.

**Figure S3.** Comparison of various parameters in the five frequent and total in‐frame deletions shown by violin plots. Logarithmic values of serum CK (A), %FVC (B), logarithmic values of plasma BNP (C), LVDd (D), and LVEF (E) levels. *p < 0.05, **p < 0.01, ***p < 0.001. BNP, brain‐derived natriuretic peptide; CK, creatine kinase; FVC, forced vital capacity; LVEF, left ventricular ejection fraction.

**Figure S4.** Comparison of various parameters without and with intervention in the five most frequent deletions and total in‐frame mutations shown by violin plots. Comparison of the logarithmic values of serum CK (A, B) and %FVC (C, D) without (0: A, C) or with (1: B, D) corticosteroids use between the initial (blue) and final (orange) surveys. Comparison of the logarithmic values of plasma BNP (E, F), LVDd (G, H), and LVEF (I, J) values without (0: E, G, I) or with (1: F, H, J) cardioprotective drugs use between the initial (blue) and final (orange) surveys. BNP, brain‐derived natriuretic peptide; CK, creatine kinase; FVC, forced vital capacity; LVEF, left ventricular ejection fraction.

**Table S1.** Background factors of study participants.
**Table S2.** Information of affected family members.
**Table S3.** Number of patients with BMD having in‐frame deletions resulting from exon‐skipping therapy.

## Data Availability

All data requests should be submitted to the corresponding author for consideration. Access to the available anonymized data may be granted following review.
