# Supplementary figures and images for: Natural history of Becker muscular dystrophy: a multicenter study of 225 patients
Source: Ann Clin Transl Neurol. 2023 Oct 26;10(12):2360–72. doi: 10.1002/acn3.51925 (PMC10723226; doi:10.1002/acn3.51925)

**A**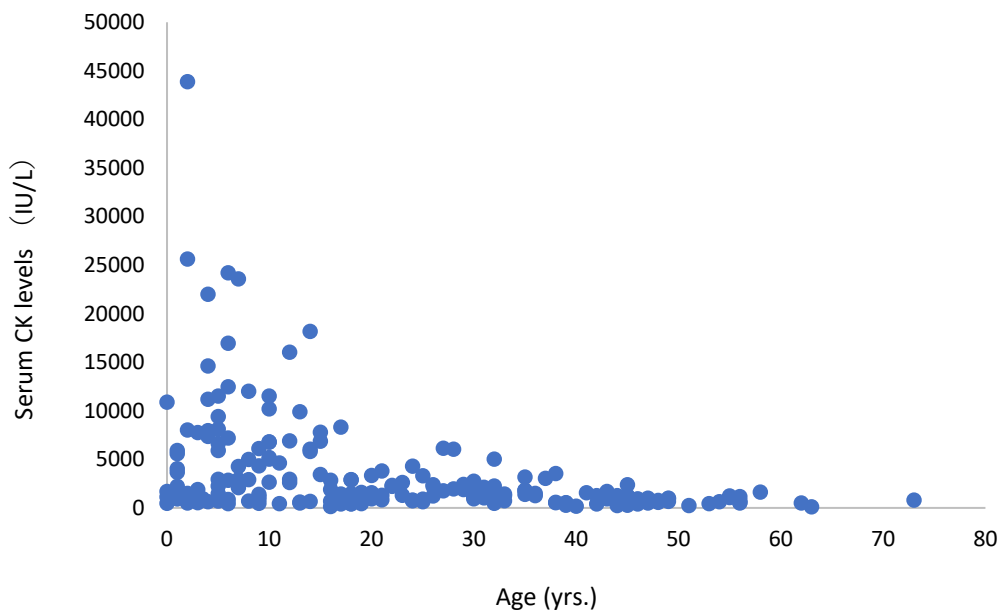**B**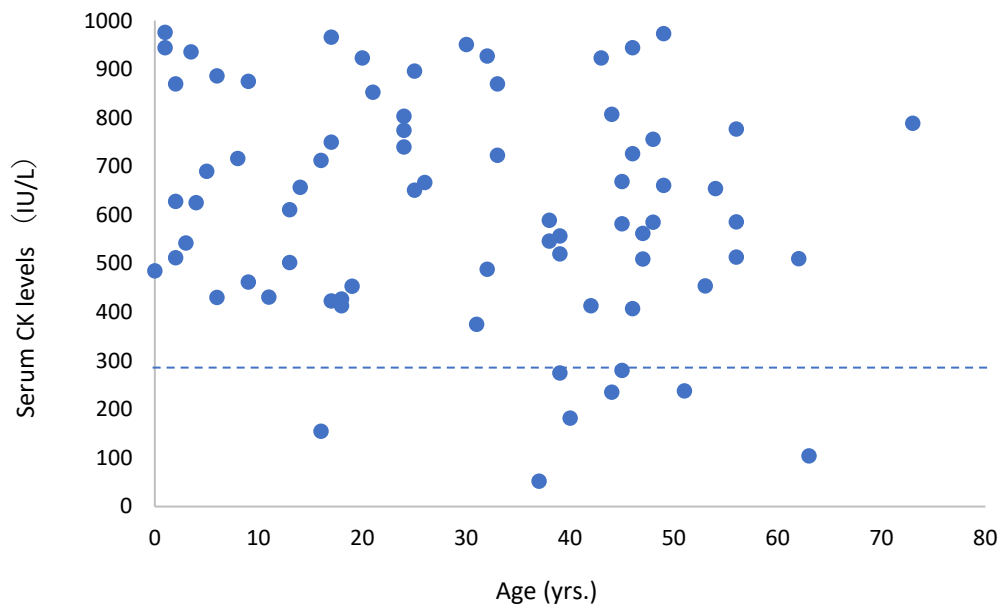**C**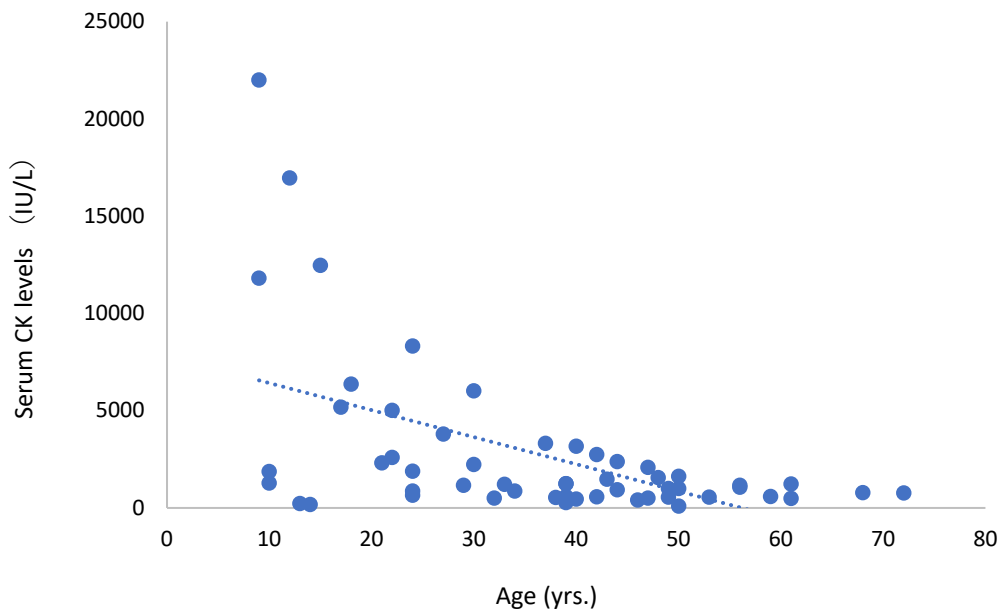

Supplement: Supplementary file 1 — Figure S1. Serum creatine kinase (CK) levels at the initial medical record survey. (A) Scatterplot showing the association between serum creatine kinase (CK) level and age at the initial survey in all participants (n = 212). (B) Scatterplot showing the association between serum CK level and age at the initial survey with cut of level of 1000 IU/L; dash line shows upper limit level of normal range (287 IU/L). (C) Scatterplot of the association between serum CK at the initial survey and age at wheelchair introduction (n = 54); dash line linear indicates the regression derived from data. [file ACN3-10-2360-s003.pdf]

**A**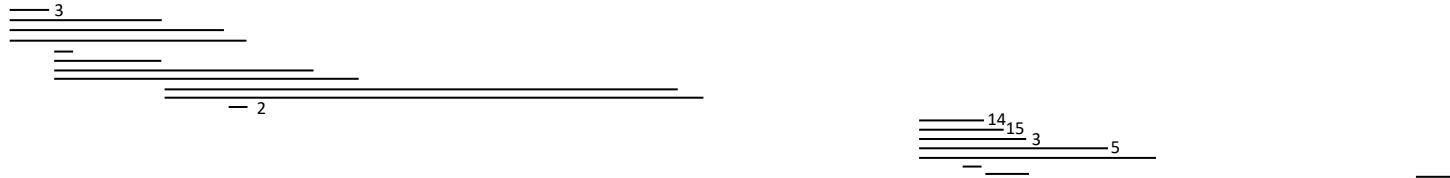**B**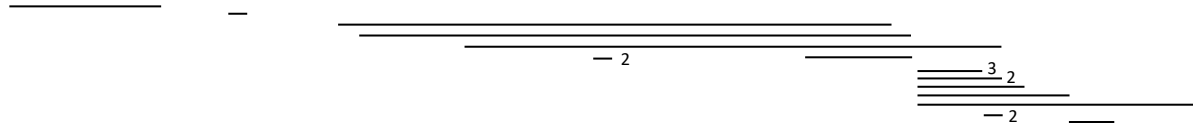**C**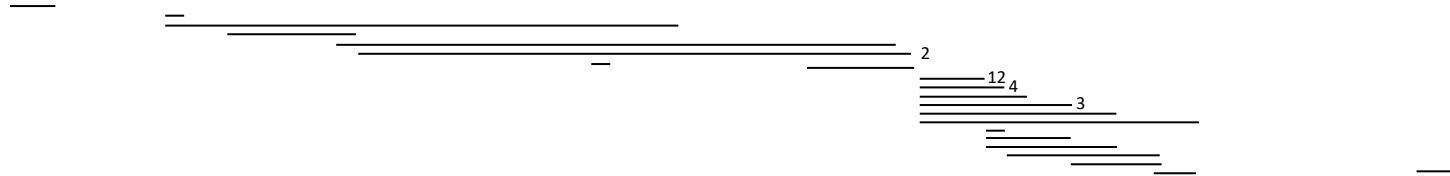

|   |   |   |   |   |   |   |   |   |    |    |    |    |    |    |    |    |    |    |    |    |    |    |    |    |    |    |    |    |    |    |    |    |    |    |    |    |    |    |    |    |    |    |    |    |    |    |    |    |    |    |    |    |    |    |    |    |    |    |    |    |    |    |    |    |    |    |    |    |    |    |    |    |    |    |    |    |    |    |
|---|---|---|---|---|---|---|---|---|----|----|----|----|----|----|----|----|----|----|----|----|----|----|----|----|----|----|----|----|----|----|----|----|----|----|----|----|----|----|----|----|----|----|----|----|----|----|----|----|----|----|----|----|----|----|----|----|----|----|----|----|----|----|----|----|----|----|----|----|----|----|----|----|----|----|----|----|----|----|
| 1 | 2 | 3 | 4 | 5 | 6 | 7 | 8 | 9 | 10 | 11 | 12 | 13 | 14 | 15 | 16 | 17 | 18 | 19 | 20 | 21 | 22 | 23 | 24 | 25 | 26 | 27 | 28 | 29 | 30 | 31 | 32 | 33 | 34 | 35 | 36 | 37 | 38 | 39 | 40 | 41 | 42 | 43 | 44 | 45 | 46 | 47 | 48 | 49 | 50 | 51 | 52 | 53 | 54 | 55 | 56 | 57 | 58 | 59 | 60 | 61 | 62 | 63 | 64 | 65 | 66 | 67 | 68 | 69 | 70 | 71 | 72 | 73 | 74 | 75 | 76 | 77 | 78 | 79 |
|---|---|---|---|---|---|---|---|---|----|----|----|----|----|----|----|----|----|----|----|----|----|----|----|----|----|----|----|----|----|----|----|----|----|----|----|----|----|----|----|----|----|----|----|----|----|----|----|----|----|----|----|----|----|----|----|----|----|----|----|----|----|----|----|----|----|----|----|----|----|----|----|----|----|----|----|----|----|----|

Supplement: Supplementary file 2 — Figure S2. Distribution of the DMD gene mutations in each phenotype. Bars represent one or more exon deletions in the DMD coding region distributed over 79 exons. (A) Left ventricular ejection fraction (LVEF) < 55% (n = 55); (B) seizures (n = 19); and (C) intellectual/developmental disability (n = 39). The numbers to the right of the bar show the number of patients. DMD, Duchenne muscular dystrophy. [file ACN3-10-2360-s004.pdf]

**A**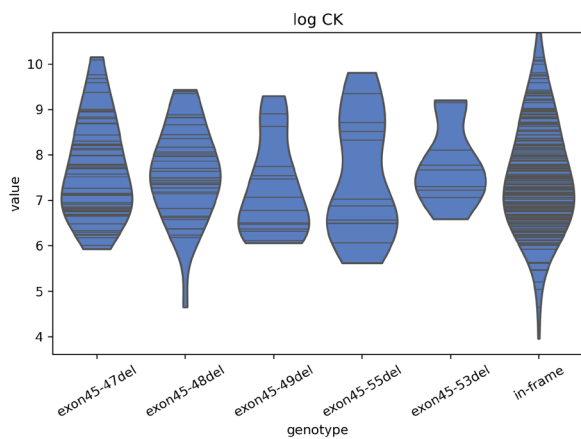**B**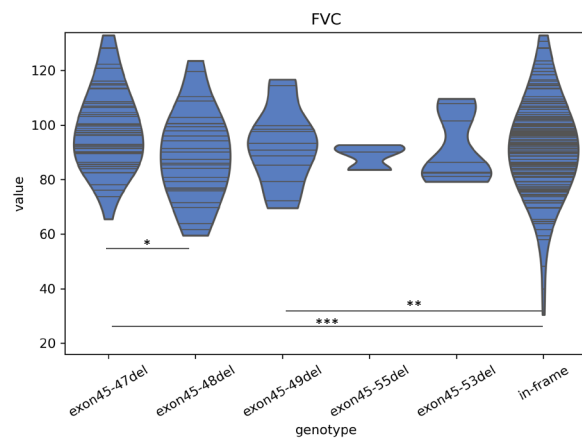**C**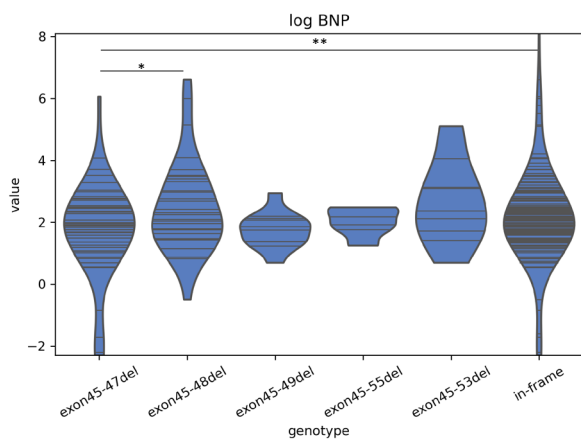**D**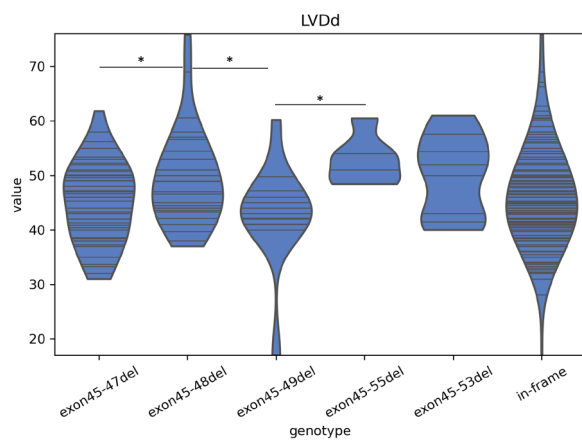**E**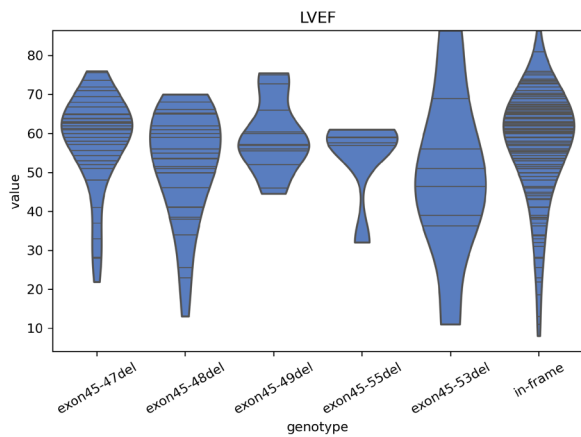

Supplement: Supplementary file 3 — Figure S3. Comparison of various parameters in the five frequent and total in‐frame deletions shown by violin plots. Logarithmic values of serum CK (A), %FVC (B), logarithmic values of plasma BNP (C), LVDd (D), and LVEF (E) levels. *p < 0.05, **p < 0.01, ***p < 0.001. BNP, brain‐derived natriuretic peptide; CK, creatine kinase; FVC, forced vital capacity; LVEF, left ventricular ejection fraction. [file ACN3-10-2360-s002.pdf]

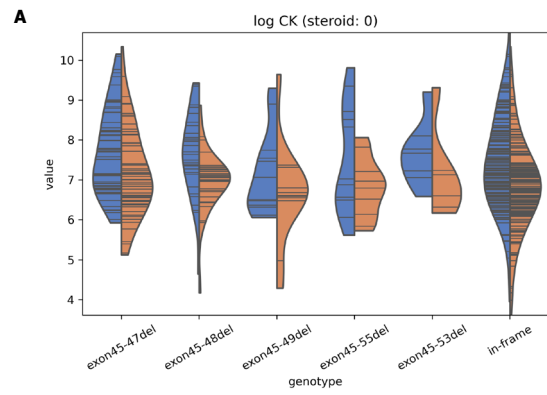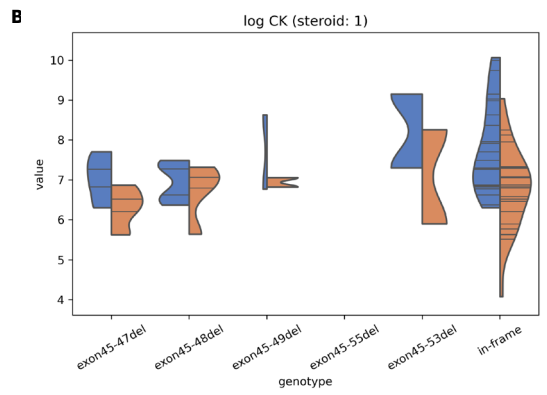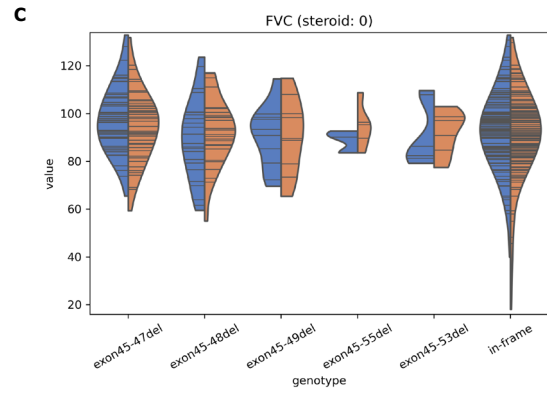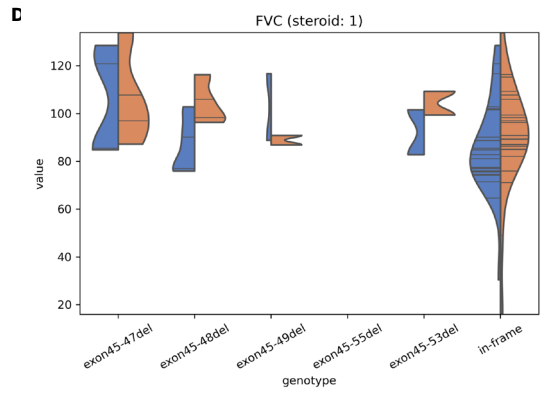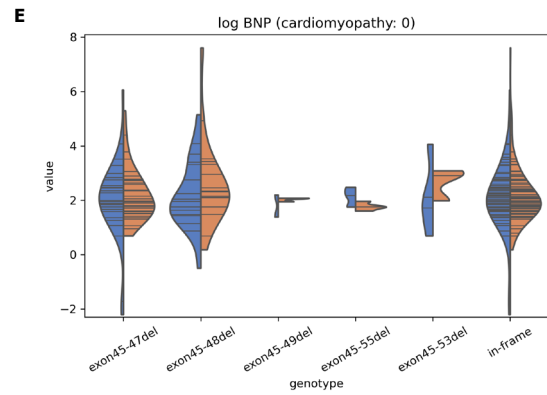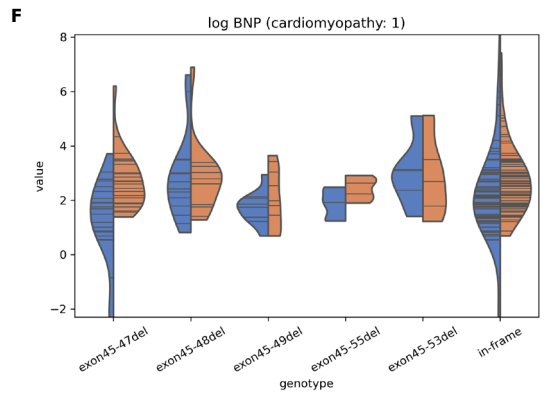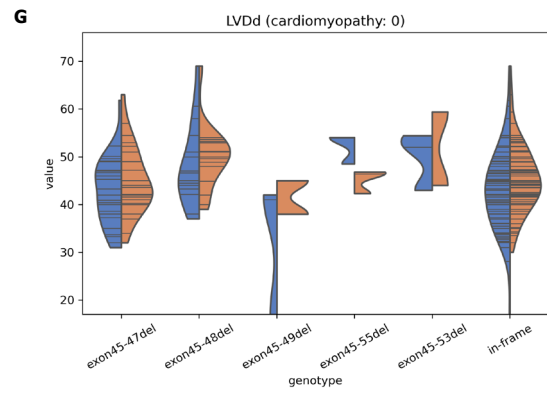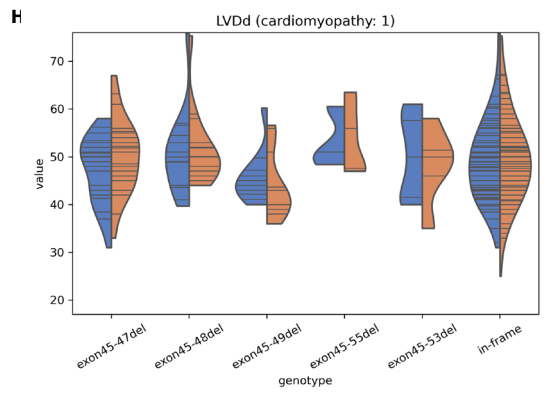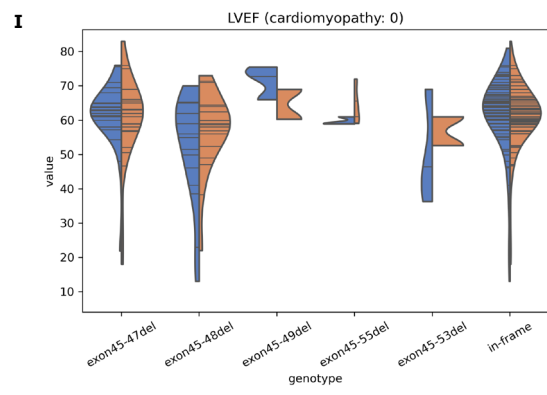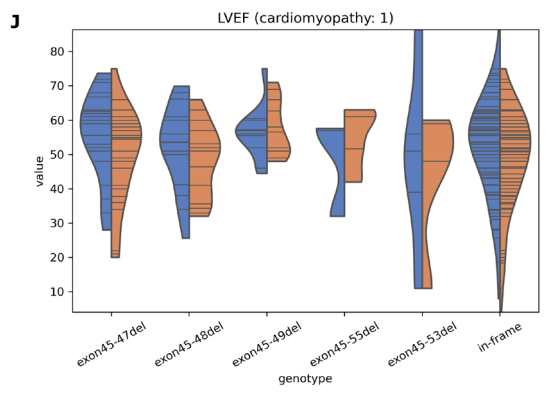

Supplement: Supplementary file 4 — Figure S4. Comparison of various parameters without and with intervention in the five most frequent deletions and total in‐frame mutations shown by violin plots. Comparison of the logarithmic values of serum CK (A, B) and %FVC (C, D) without (0: A, C) or with (1: B, D) corticosteroids use between the initial (blue) and final (orange) surveys. Comparison of the logarithmic values of plasma BNP (E, F), LVDd (G, H), and LVEF (I, J) values without (0: E, G, I) or with (1: F, H, J) cardioprotective drugs use between the initial (blue) and final (orange) surveys. BNP, brain‐derived natriuretic peptide; CK, creatine kinase; FVC, forced vital capacity; LVEF, left ventricular ejection fraction. [file ACN3-10-2360-s005.pdf]
