# Supplementary material for: Natural history of Becker muscular dystrophy: a multicenter study of 225 patients
Source: Ann Clin Transl Neurol. 2023 Oct 26;10(12):2360–72. doi: 10.1002/acn3.51925 (PMC10723226; doi:10.1002/acn3.51925)
Supplement: Supplementary file 5 — Table S1. Background factors of study participants. Table S2. Information of affected family members. Table S3. Number of patients with BMD having in‐frame deletions resulting from exon‐skipping therapy. [file ACN3-10-2360-s001.docx]

**Supplementary Tables**

**Table S1. Background factors of study participants**

|  |  | Mean ± SD (Median, Range) |
| --- | --- | --- |
| Age at earliest medical records (years) | 213 | 21.6 ± 16.6 (18, 0–73) |
| Age at registration for final enrolled patients (years) | 225 | 31.5 ± 17.8 (29, 1–81) |
| Body height (cm) | 201 | 158.0 ± 18.7 (163.4, 70.1–182.0) |
| Body weight (kg) | 199 | 52.3 ± 16.9 (55.2, 8.6–94.2) |
|  | N (%) |  |
| Initial symptoms or findings that triggered the diagnosis |  |  |
| Skeletal muscle involvement | 135 (60.0) |  |
| Slow walking or running | 49 |  |
| Myalgia, muscle cramp | 28 |  |
| Difficulty standing up and climbing stairs | 22 |  |
| Easy to trip or fall | 15 |  |
| Muscle hypertrophy | 7 |  |
| Limb weakness | 5 |  |
| Easy fatigability | 7 |  |
| Equinus | 2 |  |
| Red urine | 1 |  |
| Asymptomatic hyperCKemia | 73 (32.4) |  |
| CNS complications | 12 (5.3) |  |
| Mental retardation | 4 |  |
| Learning disability | 2 |  |
| ASD | 1 |  |
| Epilepsy | 3 |  |
| Depression | 1 |  |
| Schizophrenia | 1 |  |
| Cardiac complications | 4 (1.7) |  |
| Acute heart failure | 1 |  |
| Cardiac dysfunction | 1 |  |
| Arrhythmia | 1 |  |
| Unknown | 1 |  |
| Other comorbidities | 5 (2.0) |  |
| Hepatomegaly | 1 |  |
| Growth disturbance | 1 |  |
| Unknown | 3 |  |

SD, standard deviation; CNS, central nervous system; ASD, autism spectrum disorder

**Table S2. Information of affected family members**

| Information of affected family members | N (%) | Mean ± SD (Median, Range) |
| --- | --- | --- |
| Positive family history | 116 (51.6) |  |
| Age at onset (available) (years) | 41 (35.3) | 14.9 ± 12.9 (10, 2–45) |
| Age at wheelchair introduction (years) | 10 (13.3) | 40.4 ± 12.3 (46.5, 21–53) |
| Diagnosis |  |  |
| BMD | 71 (61.2) |  |
| NA or unknown | 45 (38.8) |  |
| Initial symptoms or findings that triggered the diagnosis |  |  |
| Motor impairment | 35 (30.2) |  |
| Myalgia | 10 (8.6) |  |
| Calf hypertrophy | 5 (4.3) |  |
| Cardiac involvement | 5 (4.3) |  |
| Intellectual/developmental disorders | 4 (3.4) |  |
| HyperCKemia | 2 (1.7) |  |
| Seizures | 2 (1.7) |  |
| Easy fatigability | 1 (0.9) |  |
| Muscle atrophy | 1 (0.9) |  |
| NA or unknown | 51 (39.3) |  |

BMD, Becker muscular dystrophy; NA, not available; SD, standard deviation

**Table S3. Number of patients with BMD having in-frame deletions resulting from exon-skipping therapy**

| Out-of-frame mutation for exon-skipping therapy | After exon skipping (in-frame mutation) | Number of patients with BMD in this study |
| --- | --- | --- |
| For exon 51 skipping |  |  |
| exon45-50del | exon45-51del | 8 |
| exon48-50del | exon48-51del | 1 |
| For exon 53 skipping |  |  |
| exon45-52del | exon45-53del | 9 |
| exon48-52del | exon48-53del | 2 |
| exon52del | exon52-53del | 1 |
| For exon 45 skipping |  |  |
| exon46del | exon45-46del | 1 |
| exon46-47del | exon45-47del | 69 |
| exon46-48del | exon45-48del | 40 |
| exon46-49del | exon45-49del | 14 |
| exon46-51del | exon45-51del | 8 |
| exon46-52del | exon45-52del | 1 |
| exon46-53del | exon45-53del | 9 |
| exon46-55del | exon45-55del | 12 |
| exon46-57del | exon45-57del | 1 |
| exon46-58del | exon45-58del | 1 |
| For exon 44 skipping |  |  |
| exon10-43del | exon10-44del | 1 |
| exon19-43del | exon19-44del | 2 |
| exon28-43del | exon28-44del | 1 |
| exon40-43del | exon40-44del | 1 |
| For exon 52 skipping |  |  |
| exon53del | exon52-53del | 1 |
| exon53-55del | exon52-55del | 1 |
| For exon 43 skipping |  |  |
| exon12-42del | exon12-43del | 1 |

This table does not cover all out-of-frame mutations targeted by each exon-skipping treatment, but the patients with BMD enrolled in this study who have the same mutations as if they were in-frame. BMD, Becker muscular dystrophy; del, deletion
